# Methodologies for Sample Multiplexing and Computational Deconvolution in Single‐Cell Sequencing

**DOI:** 10.1002/advs.202513396

**Published:** 2025-11-19

**Authors:** Yufei Gao, Weiwei Yin, Wei Hu, Wei Chen

**Affiliations:** ^1^ Department of Cardiology and Department of Cell Biology of the Second Affiliated Hospital Liangzhu Laboratory Zhejiang University School of Medicine Hangzhou Zhejiang 310012 China; ^2^ Department of Mechanical Engineering Zhejiang University Hangzhou Zhejiang 310012 China; ^3^ Key Laboratory for Biomedical Engineering of the Ministry of Education College of Biomedical Engineering and Instrument Science Zhejiang University Hangzhou Zhejiang 310012 China; ^4^ Zhejiang Provincial Key Laboratory of Cardio‐Cerebral Vascular Detection Technology and Medicinal Effectiveness Appraisal Zhejiang University Hangzhou Zhejiang China; ^5^ Kidney Disease Center The First Affiliated Hospital Zhejiang University School of Medicine Hangzhou Zhejiang 310012 China

**Keywords:** computational deconvolution, sample multiplexing, single‐cell sequencing

## Abstract

Single‐cell sequencing is revolutionizing biological research by enabling unprecedented cellular resolution, yet traditional multi‐sample experiments are often constrained by high costs and batch effects. Sample multiplexing offers a critical solution by uniquely tagging individual cells from diverse samples for pooled sequencing, thereby dramatically boosting throughput and improving data reliability by minimizing technical variability. This review provides a comprehensive and integrated perspective on the rapidly evolving field of single‐cell multiplexing. Major experimental strategies and the critical computational algorithms required for accurate sample deconvolution are surveyed, highlighting the crucial link between experimental design and computational accuracy. Furthermore, the diverse applications of these technologies in large‐scale clinical cohorts, multi‐omics integration, developmental biology, and high‐throughput drug screening are summarized. This review serves as an essential guide for researchers, empowering them to select the most appropriate methods to accelerate discoveries in disease mechanisms, therapeutic responses, and developmental biology.

## Graphical abstract

Sample multiplexing offers a critical solution for boosting throughput and minimizing batch effects in single‐cell sequencing. This review navigates the latest experimental methodologies and computational deconvolution algorithms, bridging the gap between wet‐lab design and analytical success. By summarizing cutting‐edge applications and offering practical guidance for method selection, it empowers researchers to optimize their experimental designs of single‐cell studies.

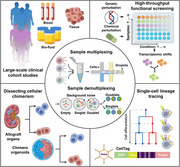

## 1 Introduction

The emergence of single‐cell sequencing technologies has profoundly transformed our understanding of human biology and disease by dissecting cellular heterogeneity with unparalleled resolution.^[^ ^1^, ^2^, ^3^, ^4^ ^]^ However, conventional multi‐sample experiments, which are typically performed in parallel, encounter substantial hurdles, such as high costs, persistent batch effects, and misleading signals from cell multiplets.^[^ ^5^, ^6^ ^]^ To overcome these constraints, sample multiplexing has become an essential and innovative approach in single‐cell genomics. This method involves the unique tagging of cells from different samples before they are pooled for unified library preparation and sequencing. Subsequently, the precise deconvolution of this pooled data relies on advanced computational tools to assign each cell back to its original sample and accurately identify multiplets.^[^ ^7^ ^]^

A variety of sample multiplexing strategies have been developed, each offering distinct benefits. These can be broadly categorized into: external sample labeling, which uses antibody‐oligonucleotide conjugates, lipid membrane insertion, or chemical reactions^[^ ^8^, ^9^, ^10^, ^11^, ^12^ ^]^; combinatorial indexing‐based multiplexing, which applies sequential barcoding during library construction^[^ ^13^, ^14^ ^]^; genetic engineering‐based cell labeling, which integrates heritable barcodes into the genome^[^ ^15^, ^16^ ^]^; and endogenous barcoding using genetic variation, which leverages natural genetic differences like single nucleotide polymorphisms (SNPs) for label‐free sample identification.^[^ ^17^ ^]^ Furthermore, hybrid methodologies that integrate experimental and genetic approaches have been developed to enhance the reliability of deconvolution^[^ ^18^, ^19^ ^]^ (Table S1, Supporting Information).

**Figure 1:**
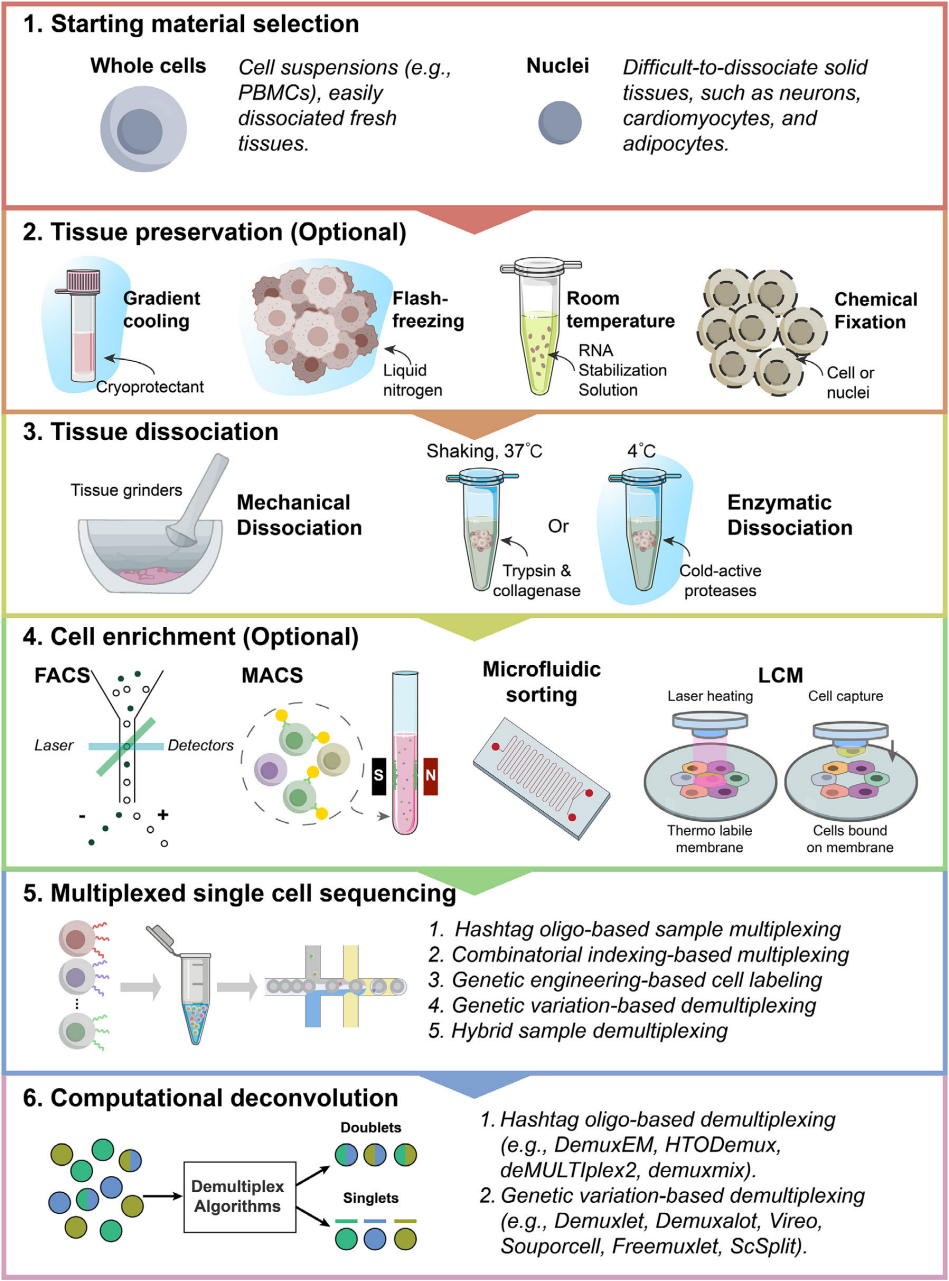
Overview of the single‐cell multiplexing workflow, from sample preparation to computational deconvolution. The workflow initiates with the selection of starting material (whole cells or nuclei), a critical decision that guides subsequent tissue preservation and dissociation strategies. Following dissociation, an optional cell enrichment step can often be performed to isolate specific cell populations and enhance statistical power for rare cell types. Prepared single‐cell suspensions are then uniquely labeled using one of several multiplexing strategies before being pooled for single‐cell sequencing. Finally, post‐sequencing, specialized computational algorithms are applied to demultiplex the pooled data, assigning each cell back to its original sample and identifying multiplets. This crucial step ensures data integrity for all downstream analyses.

The success of these experimental strategies is critically dependent on the performance of their corresponding computational demultiplexing algorithms. While tag counts ideally follow a precise bimodal distribution, real‐world data are frequently disrupted by technical noise and contamination.^[^ ^20^ ^]^ To address this, a suite of advanced algorithms has been engineered, leveraging statistical frameworks such as maximum likelihood estimation,^[^ ^21^ ^]^ negative binomial models,^[^ ^9^ ^]^ and Gaussian mixture models^[^ ^22^ ^]^ to distinguish the signal from background noise accurately. The versatility of these integrated workflows has expanded the impact of multiplexing beyond transcriptomics to diverse multi‐omics applications, including single‐cell transposase‐accessible chromatin sequencing (scATAC‐seq), proteomics, and clustered regularly interspaced short palindromic repeats (CRISPR) screening, enabling large‐scale studies that were previously unattainable.

The growing complexity and interplay between diverse experimental designs and computational algorithms underscore the need for a comprehensive overview. Such a review must not only cover the foundational principles—including single‐cell isolation, multiplexing methodologies, and computational demultiplexing algorithms—but also illuminate the critical interplay between wet‐lab design choices and downstream computational accuracy. In this review, we systematically compare state‐of‐the‐art sample multiplexing strategies and demultiplexing algorithms, placing particular emphasis on how experimental design choices impact computational performance. We summarize current applications of these methods across a broad spectrum of contexts, including clinical cohorts, multi‐omics studies, organoid and developmental biology, and high‐throughput drug screening. Furthermore, we offer a practical guide for selecting methods tailored to specific research contexts, alongside a critical assessment of the ongoing challenges in this rapidly evolving field. Our goal is to furnish the single‐cell community with an indispensable and timely resource that evaluates the current state of the art and outlines key directions for future innovation.

## 2 Sample Preparation Before Multiplexing

Upstream sample preparation is a critical first step before single‐cell multiplexing experiments. Its main purpose is to transform complex bulk tissues or biofluids into high‐quality, viable single‐cell or single‐nucleus suspensions suitable for downstream multiplexing procedures (e.g., sample pooling and cell barcoding) (Figure 1).

### 2.1 Starting Material Selection

When using whole cells as input, single‐cell RNA sequencing (scRNA‐seq) is the preferred method for obtaining the most comprehensive transcriptomic information from each cell. This approach is ideal for cell suspensions (e.g., peripheral blood mononuclear cells, PBMCs), freshly dissociated tissues, or situations where the analysis of cytoplasmic RNA is central to the biological question (**Table** **1**).

**Table 1:** Comparison of scRNA‐seq and snRNA‐seq.

Features	Single‐Cell RNA‐seq (scRNA‐seq)	Single‐Nucleus RNA‐seq (snRNA‐seq)
Cellular Input	Whole cells	Nuclei
Sample type	Fresh, intact cells	Frozen, fixed, or difficult‐to‐dissociate tissues
Isolation method	Enzymatic digestion to degrade the extracellular matrix (ECM).	Detergent lysis to dissolve the cell membrane.
Dissociation stress	High	Minimized
Transcript coverage	Full transcriptome (nuclear & cytoplasmic)	Nuclear transcripts
Experimental Flexibility	Less flexible	Highly flexible
Tissue type bias	Cell suspensions (e.g., PBMCs) can easily dissociate fresh tissues.	Difficult‐to‐dissociate solid tissues, such as neurons, cardiomyocytes, and adipocytes

By contrast, single‐nucleus RNA sequencing (snRNA‐seq), which uses isolated nuclei as input, is often more effective for capturing an unbiased representation of the cellular composition of complex tissues.^[^ ^23^ ^]^ It is well‐suited for analyzing frozen or fixed samples, making it particularly valuable for studies based on archived clinical biobanks or postmortem materials. The gentler lysis required for nuclei isolation is especially compatible with hard‐to‐dissociate cell types such as neurons, cardiomyocytes, and adipocytes,^[^ ^24^ ^]^ enabling snRNA‐seq to provide a more faithful representation of the in vivo transcriptional state.^[^ ^25^, ^26^ ^]^ Thus, snRNA‐seq is the preferred choice when the objective is to construct an unbiased cell atlas while minimizing dissociation‐induced artifacts. However, the main limitation of choosing nuclei as the starting material is that this approach only captures the transcripts within the nucleus, underrepresenting genes whose RNAs are predominantly cytoplasmic.

### 2.2 Sample Preservation Strategies

The choice of sample preservation methods for single‐cell suspensions is critical and must balance sample feasibility and experimental feasibility with the imperative of maintaining the sample's biological state. While processing fresh samples remains the gold standard for data quality, samples from large‐scale cohort studies or multi‐site studies often necessitate preservation (**Table** **2**).

**Table 2:** Overview of sample pretreatment strategies for single‐cell sequencing.

Method	Suitable sample types	Advantages	Challenges
1. Sample preservation strategies			
Fresh Processing	Fresh samples	Preserves the sample in its most natural state	Not feasible for samples from remote locations or large, staggered cohorts
Cryopreservation	Cell suspensions	Enables long‐term storage and sample biobanking	The freeze‐thaw cycle induces cell loss.
Flash‐Freezing	Solid tissues	Preserving RNA quality within the tissue context.	Ice crystal formation can cause cellular damage. Only suited for snRNA‐seq.
RNA Stabilization Solution	Cell suspensions or tissues.	Facilitates sample transportation and short‐term storage	Sacrifices cell viability and can interfere with downstream library preparation.
Chemical Fixation	Cell suspensions or tissues.	Preserving RNA integrity and cellular composition.	Compromised membrane integrity leading to mRNA leakage.
2. Tissue Dissociation Strategies			
Mechanical Dissociation	Loosely associated cells.	Preserve surface protein epitopes.	Not effective for high connective tissue.
Warm Enzymatic Dissociation	Most solid tissues.	High cell yield	Induces dissociation stress and degrades surface protein epitopes.
Cold Enzymatic Dissociation	Solid tissues	Minimizes artifacts and better preserves surface epitopes.	Reduced cell yields due to lower enzyme kinetics.
3. Cell enrichment strategies			
Density gradient centrifugation	Cell suspensions	Cost‐effective, excellent for removing debris and enriching major cell populations (e.g., PBMCs).	Labor‐intensive and time‐consuming; cannot separate cells with similar densities.
FACS	Cell suspensions	The highest‐purity complex subpopulations are isolated using multiple markers.	Shear stress reduces cell viability and induces stress artifacts.
MACS	Cell suspensions	Gentle on cells, fast, and ideal for bulk enrichment.	Limited to one or a few surface markers.
Microfluidic sorting	Cell suspensions	High throughput with low sample consumption.	Highly customized, requiring additional microfluidic devices.
PERFF‐seq	Fixed Cell suspensions.	Enrichment based on gene expression profiles.	The protocol for probe hybridization can be complex and lengthy.
LCM	Frozen or FFPE tissue sections.	Spatially Resolved	Extremely low throughput and labor‐intensive

Cryopreservation, typically utilizing dimethyl sulfoxide (DMSO) as a cryoprotectant, is the most robust and widely adopted sample preservation method.^[^ ^27^ ^]^ Its major advantage is its ability to enable batched sample processing, minimizing technical variability, especially for large cohort studies. Importantly, multiple studies have demonstrated a strong Pearson correlation (R≥0.96) between gene expression profiles of fresh and cryopreserved cells across diverse populations,^[^ ^28^ ^]^ including myeloma samples,^[^ ^29^ ^]^ microglia,^[^ ^30^ ^]^ immune cells, and cell lines.^[^ ^27^ ^]^ This makes cryopreservation the most reliable general‐purpose sample preservation strategy. However, freeze–thaw cycles in cryopreservation can induce cell loss, disproportionately affect fragile subpopulations, and trigger minor transcriptional stress responses.^[^ ^31^ ^]^

Flash‐freezing (or snap‐freezing) represents an alternative preservation strategy. It rapidly cools tissues in liquid nitrogen or on dry ice to preserve RNA and DNA integrity for single‐cell sequencing.^[^ ^32^, ^33^ ^]^ This approach halts enzymatic activity and maintains nucleic acids, but is generally unsuitable for whole‐cell analysis, as viable cells are difficult to recover after thawing. As a result, flash‐frozen samples are typically used for nuclei extraction and snRNA‐seq.

RNA stabilization solutions offer another alternative that preserves molecular integrity without freezing. These aqueous, non‐toxic reagents rapidly permeate cells and inactivate endogenous RNases, thereby protecting RNA from degradation. They enable storage and transport of bio‐samples at 4 °C for up to one week. However, they affect cell viability and may interfere with the efficiency of cell lysis or barcoding in certain scRNA‐seq protocols.^[^ ^34^ ^]^

Chemical fixation, particularly with methanol, provides a powerful but more specialized option for sample preservation.^[^ ^35^ ^]^ Its effectiveness is highly cell‐type dependent for sensitive populations such as primary neural cells. Methanol fixation can better preserve their cellular composition and minimize dissociation‐ or sorting‐induced artifacts.^[^ ^36^, ^37^, ^38^ ^]^ Although fixation can lead to some mRNA leakage, relative gene expression levels correlate well with those of fresh cells and do not appear to introduce significant technical variance.^[^ ^39^ ^]^ Other fixatives, such as formaldehyde, are mainly used for cross‐linking assays and are generally incompatible with standard 3′ or 5′ capture‐based scRNA‐seq workflows. Specialized fixatives, such as acetic‐methanol (ACME)^[^ ^40^ ^]^ and dithiobis succinimidyl propionate (DSP),^[^ ^41^ ^]^ have also been developed to improve cellular and transcriptomic integrity of fixed cells.

In summary, a variety of sample preservation strategies exist for multiplexing, with no single method being universally optimal. The choice among these methods must be tailored to the specific research purpose as well as the targeted biological system. Generally, cryopreservation offers an optimal balance of sample quality and practicality for most studies. However, for challenging applications such as neurogenomics, methanol fixation offers superior performance.^[^ ^37^ ^]^ Ultimately, robust protocols should be validated across different sample types, as demonstrated by comparative studies profiling fresh and frozen clinical tumor specimens with scRNA‐seq and snRNA‐seq.^[^ ^42^ ^]^

### 2.3 Tissue Dissociation Strategies

The method used to dissociate a solid tissue into a single‐cell suspension is among the most critical steps in a single‐cell sequencing workflow. It directly impacts cell quality and the fidelity of the resulting transcriptomic profiles. Dissociation methods generally rely on enzymatic or mechanical disruption, each with distinct trade‐offs in cell yield, cell viability, and artifact introduction (Table 2).

An optimal dissociation strategy aims to maximize cell yield while preserving biological fidelity. Warm enzymatic dissociation is the standard method for maximizing cell yield, particularly when the target cell populations are robust. The trade‐off is that it can induce significant transcriptional stress signatures, including the artificial upregulation of immediate‐early genes (IEGs, such as FOS and JUN), SOCS3, and heat‐shock proteins (HSPs), which can confound biological interpretation.^[^ ^43^ ^]^ Enzyme exposure may also cleave surface proteins, interfering with assays such as cellular indexing of transcriptomes and epitopes by sequencing (CITE‐seq).^[^ ^44^, ^45^ ^]^

Cold dissociation protocols are preferable when the goal is to capture the most authentic transcriptomic snapshot. By minimizing stress‐induced artifacts, these methods provide expression profiles that more accurately reflect in vivo states. Systematic evaluations have shown that digestion performed on ice or with cold‐active proteases avoids the strong stress responses observed in standard warm (37 °C) protocols.^[^ ^46^, ^47^ ^]^ Cold dissociation is therefore especially valuable when studying stress‐sensitive pathways, subtle transcriptional changes, or preserving unbiased cellular representation.

In practice, the optimal tissue dissociation strategy is usually a careful combination of gentle mechanical disruption followed by a tailored enzymatic cocktail at the appropriate temperature, guided by the specific tissue type and the study's scientific question. Michal et al. provided a systematic toolbox for processing fresh and frozen tumors from eight distinct tumor types using scRNA‐Seq and snRNA‐Seq, as valuable references for subsequent tumor research.^[^ ^42^ ^]^ Similarly, many published sample processing protocols have been compiled, which can be accessed from the website, such as “http://www.worthington‐biochem.com/tissuedissociation/default.html”.

### 2.4 Cell Enrichment Strategies

Following tissue dissociation, a cell enrichment procedure is often necessary for multiplexing rare cell types or a predefined population of particular interest. The choice of methods often involves a careful consideration of population purity, cell viability, sample throughput, and the preservation of the cells' native transcriptional state (Table 2).

Density gradient centrifugation is a classic and efficient approach that separates cells based on density differences. It is widely used to sort specific cell populations from fresh clinical samples, such as PBMC from peripheral blood, the four central nervous cell types from human fetal tissue, and hematopoietic stem cells from bone marrow.^[^ ^48^, ^49^ ^]^

Fluorescence‐activated cell sorting (FACS) remains the gold standard when the high population purity (95–100%) and precise isolation of complex subpopulations are required.^[^ ^50^ ^]^ FACS offers fast and unparalleled multi‐parametric resolution, enabling sorting based on both surface and intracellular protein expression.^[^ ^51^ ^]^ As a result, it can achieve precise sorting (or enrichment) of subtle subpopulations. Its main limitation is the need for large input cell numbers and the potential shear stress exerted on single cells flowing through the chamber, which can reduce cell viability.

Magnetic‐activated cell sorting (MACS) provides a gentler alternative, particularly when cell viability and the preservation of the native transcriptional state are priorities. It is well‐suited for the fast and bulk enrichment of a defined cell population, often achieving purity exceeding 90%.^[^ ^52^ ^]^ This property makes it well‐suited for applications where cells are destined for downstream functional assays, cell culture, or when the scientific question is highly sensitive to stress‐induced gene expression. The primary trade‐off is that MACS is generally limited to positive or negative selection on a few markers and cannot distinguish cells based on varying expression levels.

Microfluidic sorting has emerged as a transformative technology, exploiting affinity‐based capture, immunomagnetic separation, or label‐free physical parameters such as size, deformability, or dielectric properties.^[^ ^53^, ^54^ ^]^ These platforms integrate sorting and handling in miniaturized devices, enabling high throughput with low sample input, and are increasingly important in single‐cell studies.

Programmable enrichment via RNA FlowFISH by sequencing (PERFF‐seq) enables the enrichment of cells based on intracellular transcript abundance detected with fluorescent probes.^[^ ^55^ ^]^ This allows the isolation of rare types or states defined by gene expression rather than surface markers. Although powerful, it requires fixation, permeabilization, and a relatively complex probe hybridization protocol.

Laser capture microdissection (LCM) is uniquely suited when the spatial context within the tissue microenvironment must be preserved.^[^ ^56^ ^]^ LCM offers unmatched precision in isolating specific cells or clusters directly from tissue sections, making it ideal for linking a cell's molecular profile to its precise physical location and morphology. However, it is limited by extremely low throughput, reliance on expert pathological annotation, and potential artifacts during tissue preparation.

## 3 Sample Multiplexing Methodologies

### 3.1 Hashtag Oligo‐Based Sample Multiplexing

**Figure 2:**
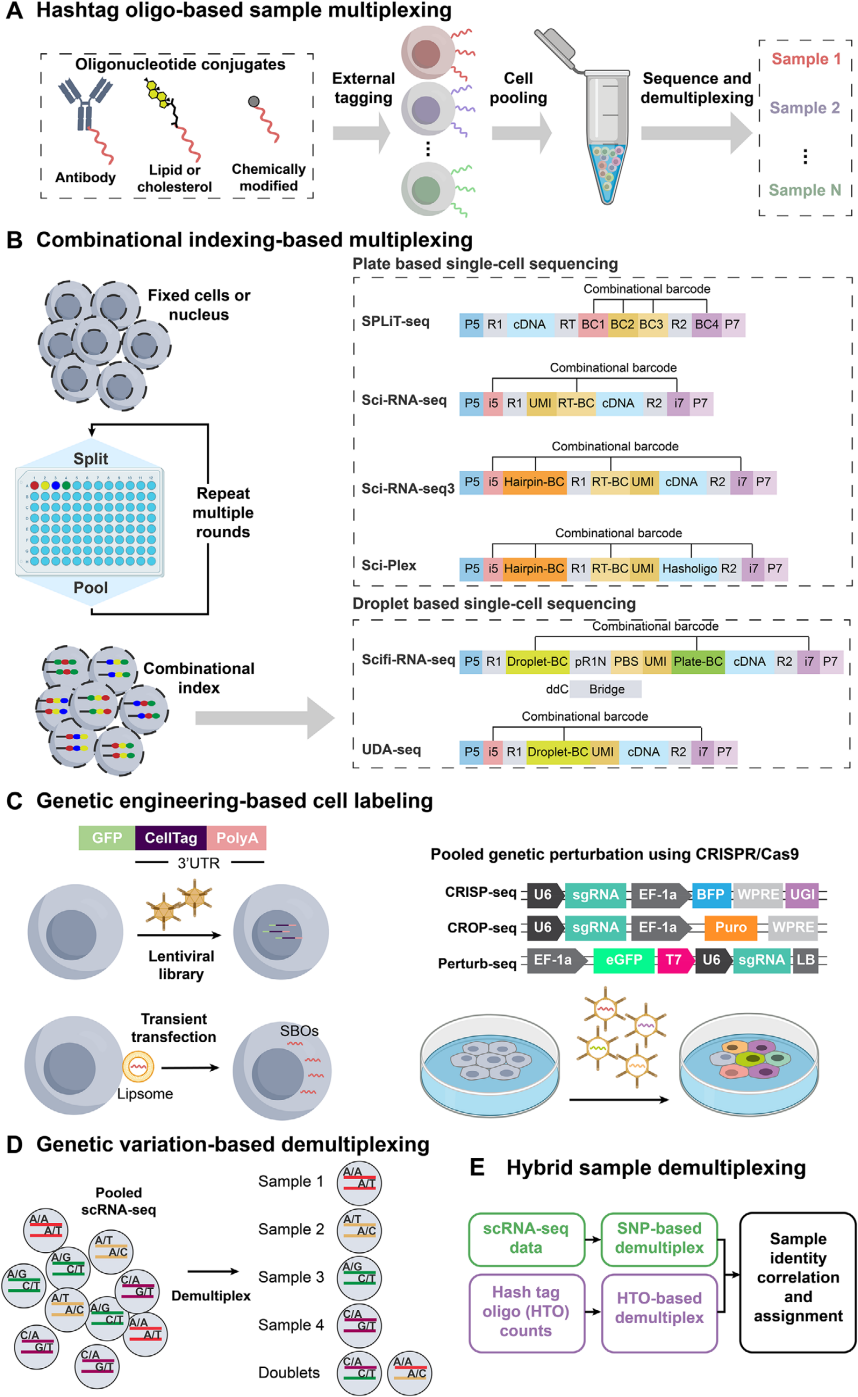
Schematic overview of sample multiplexing strategies for single‐cell sequencing. A) Cells or nuclei from distinct samples are labeled with unique DNA oligonucleotide tags, known as hashtag oligonucleotides (HTOs). These tags can be conjugated to antibodies targeting surface proteins, anchored into the cell membrane via lipid modification, or attached through direct chemical reactions. B) Millions of unique barcode combinations are generated through combinatorial indexing, enabling ultra‐high‐throughput and cost‐effective sequencing. C) Unique DNA barcodes are integrated into the cell's genome using methods such as transient transfection, lentiviral vectors (e.g., CellTag), or CRISPR/Cas9 systems (e.g., CROP‐seq, Perturb‐seq). D) Genetic variation‐based demultiplexing is a label‐free approach that identifies cell origins by leveraging naturally occurring genetic differences, such as SNPs, among distinct individuals. E) Hybrid sample demultiplexing synergistically combines multiple strategies, such as cell hashing and SNP‐based deconvolution, to enhance overall accuracy and robustness.

Hashtag oligo‐based sample multiplexing is a strategy where cells or nuclei from distinct samples are labeled with unique DNA oligonucleotide tags, known as hashtag oligonucleotides (HTOs). This enables the samples to be pooled for a single‐cell analysis workflow. Subsequent computational demultiplexing assigns each cell's profile back to its sample of origin, a strategy that substantially reduces costs, minimizes batch effects, and increases throughput (**Figure** **2**A).

#### 3.1.1 Antibody‐Based Sample Labeling

Antibody‐based sample labeling utilizes antibodies to target specific protein epitopes, with a covalently attached DNA oligonucleotide that serves as a readable barcode for sequencing. This approach is broadly known as cellular indexing of transcriptomes and epitopes by sequencing (CITE‐seq). For sample multiplexing, each antibody is conjugated to a unique HTO, which effectively tags cells from a specific sample or experimental condition. This allows multiple samples to be pooled and processed in a single microfluidic run, which not only dramatically increases throughput but also minimizes technical batch effects.^[^ ^8^, ^9^ ^]^ RNA expression and protein sequencing (REAP‐seq)^[^ ^57^ ^]^ expands the capability by measuring up to 82 proteins alongside over 20 000 genes. Single‐cell integrated transcriptome and oligonucleotide sequencing (SCITO‐seq)^[^ ^58^ ^]^ leverages combinatorial indexing of DNA‐barcoded antibodies on microfluidic platforms, achieving ultra‐high‐throughput profiling of more than 100,000 cells per reaction.

The utility of antibody‐oligonucleotide conjugates extends beyond live‐cell transcriptomics to isolated nuclei and integrated multi‐omic assays. To enable multiplexing for snRNA‐seq, a strategy known as “Nucleus Hashing” uses antibodies that recognize nuclear pore complex proteins to label nuclei from different samples.^[^ ^21^ ^]^ This approach, also termed NuHash, has been adapted for single‐nucleus ATAC‐seq (snATAC‐seq) as well.^[^ ^59^ ^]^ However, a key limitation is the potential degradation of these nuclear proteins during harsh tissue dissociation protocols, which can reduce labeling efficiency.

The modularity of the technology has also allowed for its seamless integration into complex multi‐omic workflows. For example, ECCITE‐seq can simultaneously detect surface proteins, immune clonotypes (T‐cell receptor (TCR); B‐cell receptor (BCR)), and single guide RNAs (sgRNAs) for CRISPR screens from the same cell.^[^ ^60^ ^]^ Similarly, ASAP‐seq and DOGMA‐seq concurrently profile chromatin accessibility, protein levels, and mitochondrial DNA.^[^ ^61^ ^]^ More recent developments like PHAGE‐ATAC leverage engineered nanobodies to measure protein levels and chromatin accessibility simultaneously,^[^ ^62^, ^63^ ^]^ further demonstrating the versatility of the technology.

Successful demultiplexing of data generated with antibody‐oligonucleotide conjugates requires careful protocol optimization, hinging particularly on three key experimental factors. First, sample quality is paramount. Specifically, high cell viability must be maintained, as dead cells can non‐specifically bind antibodies and thereby increase background noise.^[^ ^64^ ^]^ For immune cell populations, pre‐treatment with an Fc‐blocking reagent is essential to mitigate non‐specific, receptor‐mediated antibody binding.^[^ ^65^ ^]^ Second, antibody titration is arguably the most crucial step in optimization. An insufficient antibody concentration results in a poor signal‐to‐noise ratio, whereas excessive antibody concentrations inflate background noise and sequencing costs without any corresponding improvement in data quality.^[^ ^45^ ^]^ Although flow cytometry can serve as a proxy, method‐specific optimization is often necessary to determine the optimal concentration for CITE‐seq applications, as demonstrated by studies on human PBMCs.^[^ ^66^ ^]^ Finally, for solid tissues, the enzymatic dissociation process must be carefully controlled. Proteolytic enzymes can cleave cell surface epitopes, thereby reducing or eliminating antibody binding sites. Consequently, the choice of enzymes and digestion duration must be meticulously optimized to balance dissociation efficiency with the preservation of protein epitopes.^[^ ^67^ ^]^

#### 3.1.2 Lipid‐Based Sample Labeling

MULTI‐seq is a cost‐effective and high‐throughput multiplexing method that utilizes lipid‐modified oligonucleotides (LMOs) or cholesterol‐modified oligonucleotides (CMOs) to label cells and nuclei for single‐cell and single‐nucleus RNA sequencing.^[^ ^10^, ^68^ ^]^ These tagged indices spontaneously anchor into the cell membrane, providing a universal, non‐perturbative method for barcoding samples. The technique is compatible with standard upstream protocols like enzymatic dissociation and cryopreservation and has been optimized to reduce both experimental costs and batch effects in multiplexed analyses.^[^ ^69^ ^]^

Benchmarking studies comparing lipid‐based and antibody‐based hashing have revealed distinct, context‐dependent strengths. While antibody hashing excels with intact cells, especially in clinical samples such as PBMCs, lipid hashing is particularly effective for isolated nuclei and for specific tissues such as the brain^[^ ^70^ ^]^ (**Table** **3**). This highlights that the optimal choice of multiplexing strategy is critically dependent on both the sample type (cells versus nuclei) and tissue of origin.

**Table 3:** Comparison of hashtag oligo‐based sample multiplexing methods across sample types (The data is derived from publicly available literature).

References	Multiplexing Method	Principle	Sample type	Reported hashing accuracy	Reported mislabeling rate
Mylka V., et al., Genome Biology, 2022.^[^ ^70^ ^]^	TotalSeq‐A / ‐C	Antibody‐based sample labeling	Cell lines	0.91 – 0.96	0.1% – 0.18%
			PBMCs	0.82 – 0.84	1.53%
			Nuclei	Low (0.50 – 0.51)	3.8% – 4.8%
	CellPlex (CMO)	Lipid‐based sample labeling	PBMCs	0.93	Not Reported
			Nuclei	0.84	Not Reported
	MULTI‐seq (LMO)		Cell Lines	0.84	0.89%
			Tissue	0.78	Not Reported

#### 3.1.3 Chemically‐Bonded Sample Labeling

Chemically modified oligonucleotides provide a flexible method compatible with both live and fixed cells, overcoming the limitations of antibody‐based approaches that depend on surface antigen expression. Gehring et al.^[^ ^12^ ^]^ developed a one‐pot, two‐step reaction to chemically attach unique DNA oligonucleotides to cells, precipitate oligos onto the cells, enabling tagging across various organisms without requiring specific epitopes or genetic modifications. This approach is ideal for large‐scale perturbation experiments, as shown in a 96‐plex study with the neural stem cells (NSCs). Fang et al.^[^ ^71^ ^]^ used Concanavalin A to conjugate biotinylated single‐strand DNA (ssDNA) barcodes to cells or nuclei, making it compatible with both scRNA‐seq and snATAC‐seq for integrated transcriptomic and epigenomic analysis.

#### 3.1.4 Deconvolution Tools Based on Hashtag Oligo

**Figure 3:**
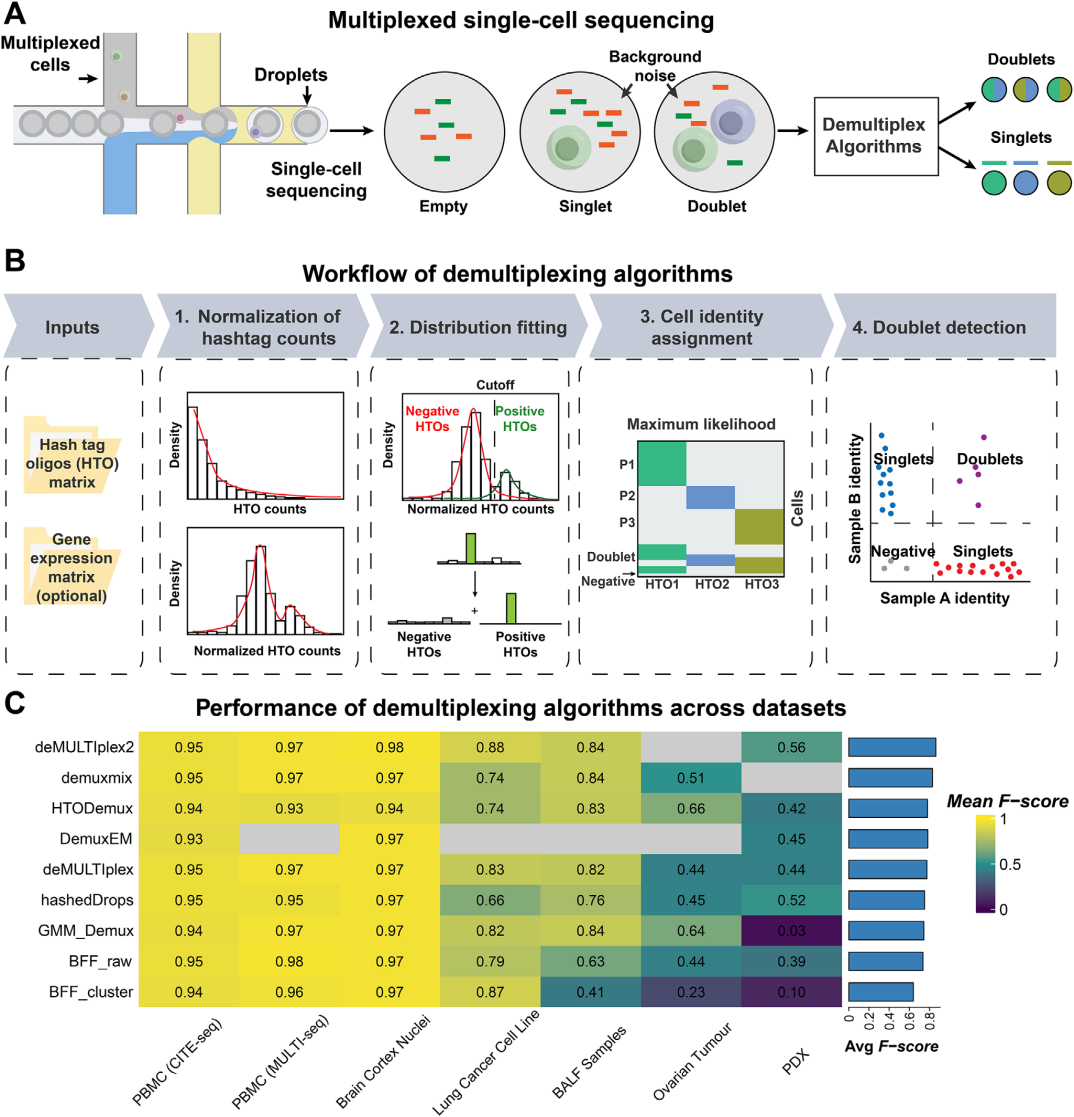
The computational framework for hashtag oligo‐based demultiplexing. A) Accurate sample demultiplexing must overcome two primary technical hurdles: the reliable identification of doublets (multiple cells captured in a single droplet) and the removal of background noise from sources like unbound HTOs, which can cause false cell assignments. B) The standard demultiplexing workflow begins by normalizing raw HTO counts. A statistical distribution model (e.g., a Gaussian or bimodal model) is then fitted to these counts to distinguish true positive signals from background noise. Based on this model, each cell is assigned to its most probable sample of origin and classified as a singlet, doublet, or unassigned. C) This panel summarizes the published benchmark of deconvolution tools across seven datasets, representing diverse sample types (e.g., PBMCs, BALF: bronchoalveolar lavage fluid, ovarian tumors, and PDX: patient‐derived xenograft).^[^ ^20^, ^78^ ^]^ Performance is quantified by the *F‐score*. Detailed information on the datasets is provided in Table S2 (Supporting Information).

Accurate sample demultiplexing is essential for single‐cell analyses but is complicated by several technical challenges. Issues such as suboptimal tag binding, the persistence of unbound HTOs, and tag cross‐contamination during pooling can generate a significant background of “false” HTO counts (**Figure** **3**A). This noise complicates the differentiation of true signals that indicate a cell's sample origin from the background, making robust computational methods necessary.

The standard computational framework for demultiplexing begins by normalizing raw HTO counts to distinguish positive signals from background noise, often using distribution models like Gaussian or bimodal fitting. Based on this, cells are classified as singlets, doublets, or unassigned (Figure 3B).

A variety of specialized algorithms employing robust statistical frameworks have been developed to overcome the challenges of demultiplexing (Table S2, Supporting Information).^[^ ^20^, ^72^ ^]^ HTODemux, a widely used tool that integrates with the Seurat package, relies on k‐medoid clustering and a negative binomial distribution to separate signals in standard applications.^[^ ^9^ ^]^ Similarly, Demuxmix combines k‐means clustering with a negative binomial mixture model to make probabilistic assignments.^[^ ^73^ ^]^

Several tools utilize sophisticated mixture models to statistically distinguish signal from noise. For instance, DemuxEM improves multiplet detection by estimating background noise from empty droplets, which helps correct for ambient RNA contamination.^[^ ^21^ ^]^ A common approach, employed by both GMM‐Demux and scDemultiplex, is to use Gaussian mixture models (GMMs) to compute the posterior probability of a cell's assignment, providing a confidence metric for its singlet or multiplet status.^[^ ^22^, ^74^ ^]^ Similarly, HashSolo applies a Bayesian method that models the overall HTO count distribution as a mixture of two log‐normal distributions, representing signal and noise, respectively.^[^ ^75^ ^]^

Other algorithms focus on setting optimal classification thresholds or explicitly modeling sources of noise. dMULTIplex uses Gaussian kernel density estimation for this purpose, aiming to maximize singlet recovery.^[^ ^10^ ^]^ For datasets with strong signals, hashedDrops offers an intuitive approach by classifying cells based on simple log‐fold changes.^[^ ^76^ ^]^ More advanced methods like BFF (BFF_raw/BFF_cluster) apply bimodal quantile normalization alongside Gaussian kernel density to robustly identify cell populations.^[^ ^77^ ^]^ For large and noisy datasets, deMULTIplex2 excels by explicitly modeling tag cross‐contamination using generalized linear models (GLM) and expectation‐maximization (EM) algorithms.^[^ ^78^ ^]^

Systematic benchmarks of HTO demultiplexing methods demonstrate that sophisticated clustering‐based methods (e.g., demuxmix, HTODemux) and expectation‐maximization (EM)‐based methods (e.g., deMULTIplex2, DemuxEM) consistently deliver the most robust performance across diverse datasets and tagging chemistries.^[^ ^72^, ^78^ ^]^ In contrast, methods that assume a clear bimodal HTO distribution, such as BFF (BFF_raw/BFF_cluster), often struggle with lower‐quality data, as this assumption frequently does not hold (Figure 3C; Table S2, Supporting Information). These findings are broadly consistent across different HTO tagging protocols (e.g., antibody‐ and lipid‐based) and tissue types, underscoring that algorithm performance is largely independent of the specific barcoding chemistry. Crucially, since HTO data quality is uncorrelated with RNA data quality, selecting a robust demultiplexing algorithm is essential for maximizing the recovery of cells from datasets with even suboptimal HTO signals.

Normalization is also a critical determinant of accurate demultiplexing, yet many tools overlook the impact of sequencing depth variability.^[^ ^20^ ^]^ Benchmarking studies have shown that combining per‐cell centered log‐ratio (CLR) normalization^[^ ^8^ ^]^ with a clustering‐based assignment offers the most robust performance, especially for noisy data. This approach leverages the compositional nature of hashtag counts and avoids making assumptions about noise distributions. Its primary limitation lies in suboptimal performance in doublet detection, suggesting that an optimal workflow should therefore integrate this normalization and clustering strategy with post hoc doublet filtering based on RNA data.

To streamline tool selection, comprehensive platforms such as cellHashR have been developed.^[^ ^77^ ^]^ These resources provide a unified interface to run and compare multiple demultiplexing algorithms, empowering researchers to efficiently identify the most suitable method for their specific experimental needs.

### 3.2 Combinational Indexing‐Based Multiplexing

Combinatorial indexing generates unique, cell‐specific barcodes during library preparation through a series of “split‐pool” steps (Figure 2B). In this approach, a population of cells or nuclei is repeatedly split into separate reaction wells, where a unique DNA barcode is added in each round. The population is then pooled back together before the next split‐pool cycle. This process creates millions of unique barcode combinations, allowing each cell to be individually identified in a massively parallel fashion.

This strategy was first established for large‐scale transcriptomics. SPLiT‐seq (split‐pool ligation‐based transcriptome sequencing) employs four rounds of split‐pool barcoding to generate over 21 million barcode combinations, enabling the profiling of over 150,000 single‐nucleus transcriptomes in a single experiment.^[^ ^79^ ^]^ A parallel development, single‐cell combinatorial indexing RNA sequencing (sci‐RNA‐seq), initially used a two‐round strategy involving in situ reverse transcription and Tn5 tagmentation.^[^ ^80^ ^]^ This was later advanced to sci‐RNA‐seq3, which incorporated a third indexing round to improve nuclei recovery and data quality.^[^ ^81^, ^82^ ^]^ Building on this, sci‐Plex adapted the method for high‐throughput chemical screens by pre‐marking nuclei with polyadenylated oligonucleotide, enabling high‐throughput chemical screens at up to 5000‐plex.^[^ ^83^ ^]^

To overcome the scalability limitations of traditional droplet‐based methods, combinatorial indexing has been integrated with microfluidic workflows. Single‐cell combinatorial fluidic indexing RNA‐seq (scifi‐RNA‐seq) utilizes a single, upstream pre‐indexing step before droplet encapsulation. This hybrid approach maintains the advantages of droplet‐based isolation while enabling the cost‐effective sequencing of millions of cells.^[^ ^84^ ^]^ This concept has been extended to chromatin profiling with methods like scifi‐ATAC‐seq and dsciATAC‐seq, which use barcoded Tn5 transposase for bulk barcoding of open chromatin before droplet encapsulation, facilitating high‐throughput analysis of up to 100 000 cells per experiment.^[^ ^85^, ^86^ ^]^

The power of combinatorial indexing is further demonstrated in its application to multi‐omics. Sci‐CAR and Paired‐seq integrate sci‐ATAC‐seq and sci‐RNA‐seq to jointly profile chromatin accessibility and mRNA from the same single cells at a massive scale.^[^ ^87^, ^88^ ^]^ Similarly, SUM‐seq and SNuBar‐ATAC enable ultra‐high‐throughput co‐assays of chromatin accessibility and gene expression in single nuclei.^[^ ^89^, ^90^ ^]^ More recent platforms such as UDA‐seq and OAK further combine droplet microfluidics with combinatorial indexing to achieve ultra‐high‐throughput multimodal single‐cell profiling.^[^ ^91^, ^92^ ^]^

The success of combinatorial indexing methods hinges on the meticulous optimization of several key experimental parameters. First, high‐quality starting material is critical, as intact, debris‐free nuclei are required to prevent aggregation and preserve RNA integrity throughout the complex, multi‐step protocol. A central challenge lies in managing the doublet rate, which must be carefully controlled by balancing cell loading density against the complexity of the barcode library at each split–pool step.^[^ ^13^ ^]^ Furthermore, the efficiency of each sequential enzymatic reaction, such as reverse transcription and ligation, is crucial, as cumulative sample loss during numerous washing and handling steps can significantly compromise the final cell yield. For specialized applications such as sci‐ATAC‐seq, additional techniques like lithium‐assisted nucleosome depletion (LAND) or sodium dodecyl sulfate (SDS) crosslinking are employed to remove DNA‐bound nucleosomes while maintaining nuclear integrity—an essential prerequisite for accurate open chromatin profiling.^[^ ^85^, ^87^, ^93^ ^]^

### 3.3 Genetic Engineering‐Based Cell Labeling

Genetic engineering involves the stable integration of unique DNA barcodes into a cell's genome, providing a permanent and heritable mark for identification. This approach is particularly powerful for lineage tracing and long‐term studies (Figure 2C).

One straightforward method involves the universal transient transfection of cells with short barcode oligonucleotides (SBOs), which enables high‐throughput profiling of transcriptional responses to various perturbations, such as drug treatments.^[^ ^15^ ^]^ For more stable, long‐term labeling, CellTag indexing utilizes lentiviral vectors to integrate barcodes, a strategy ideal for in vivo and in vitro studies of cell differentiation and lineage commitment.^[^ ^16^, ^94^ ^]^ This technique has been extended to multi‐omics with advancements like CellTag‐multi, which integrates barcoding with scATAC‐seq for simultaneous lineage tracing and chromatin accessibility profiling.^[^ ^95^ ^]^

By combining barcoding with CRISPR/Cas9 genome editing, this approach enables massively parallel functional genomic screens at single‐cell resolution. These methods link specific genetic perturbations (via guide RNAs, or gRNAs) to their direct transcriptional consequences in each cell. Early methods like CRISP‐seq used barcoded lentiviral vectors containing both a gRNA and a unique guide index (UGI) to link perturbations to scRNA‐seq profiles.^[^ ^96^ ^]^ This was further streamlined by CROP‐seq, which repurposed the gRNA sequence itself as an identifiable barcode.^[^ ^97^ ^]^ The Perturb‐seq platform expanded the scale of these screens by employing pooled lentiviral libraries with distinct gRNAs and guide barcodes (GBCs) for large‐scale profiling.^[^ ^87^ ^]^ This powerful framework has since been adapted for multi‐omic readouts, as demonstrated by Multiome Perturb‐seq, which simultaneously measures the impact of perturbations on both gene expression and chromatin accessibility.^[^ ^98^ ^]^

The success of genetic engineering‐based cell labeling is critically dependent on the precise control of several key experimental parameters. For lineage tracking, the multiplicity of infection (MOI) must be carefully titrated to a low level (e.g., ≈0.1) to ensure most cells receive a single, unique barcode, thereby preventing confounding lineage assignments while maintaining sufficient labeling efficiency.^[^ ^99^ ^]^ Equally crucial is the vector design, which must facilitate robust barcode expression from a highly complex library without perturbing cellular function. Finally, the transfection or transduction efficiency of the target cell type is a key determinant, as low efficiency, particularly in primary cells, can pose a significant practical limitation.^[^ ^100^ ^]^

### 3.4 Genetic Variation‐Based Demultiplexing

Genetic variation‐based demultiplexing is a label‐free strategy that leverages inherent genetic differences—primarily SNPs—among pooled samples to computationally assign each cell to its individual of origin (Figure 2D). This approach requires no external labeling and is also effective for detecting cell multiplets. These methods are broadly divided into two categories: those that require a reference genotype for each sample and those that are “genotype‐free.”

Methods that require a reference genotype map single‐cell reads to a list of known SNPs from pre‐existing genotyping data to determine each cell's origin. Demuxlet pioneered this approach, using a SNP‐based mixture model and maximum likelihood estimation (MLE) to assign cell identities.^[^ ^17^ ^]^ It can achieve high accuracy (e.g., 97% singlet assignment) with as few as 50 SNPs per cell. Its performance was validated in a study of PBMCs from lupus patients, where it correctly identified over 99% of singlets. Building on this, Demuxalot improves computational efficiency by focusing on the most informative genetic variants, making the method scalable for large cohorts.^[^ ^101^ ^]^ For datasets with sparse coverage or low RNA content, scSNPdemux provides a more sensitive alternative for cell assignment.^[^ ^102^ ^]^ Beyond nuclear SNPs, mitochondrial RNA (mtRNA) variants can also serve as powerful endogenous barcodes. Tools such as mitoSplitter and MitoSort have demonstrated the utility of this approach for rapidly demultiplexing large datasets.^[^ ^103^, ^104^ ^]^

To overcome the limitation of requiring external genotyping data, genotype‐free approaches infer sample identities directly from the pooled single‐cell sequencing data. Vireo employs a Bayesian model with variational inference to demultiplex scRNA‐seq data, achieving high accuracy even with low coverage, though its performance can decline in pools with a large number of samples.^[^ ^105^ ^]^ scSplit uses a hidden state model for accurate demultiplexing, while Souporcell applies a sparse mixture model with a deterministic annealing EM algorithm to cluster cells by genotype, a method that also estimates ambient RNA contamination.^[^ ^106^, ^107^ ^]^ To streamline the use of these tools, Demuxafy integrates multiple algorithms into a single, user‐friendly platform with optimized ensembles.^[^ ^108^ ^]^

Systematic benchmarks of genetic variation‐based demultiplexing methods on challenging simulated datasets have identified Demuxalot as the top‐performing tool, offering an optimal balance of high accuracy, computational efficiency, and usability^[^ ^108^, ^109^ ^]^ (**Table** **4**). Vireo and Souporcell also demonstrate strong performance, particularly in reference‐free scenarios. In contrast, scSplit consistently underperforms, hampered by low accuracy and prohibitive computational requirements (often taking multiple days).

**Table 4:** Benchmarking genetic variation‐based demultiplexing methods using mean F‐score (The data is derived from publicly available literature).

References	Sample type	Method Name	Principle	Mean F‐score	Mean time consumption (h)	Mean memory consumption (GB)
Neavin D et al., Genome Biology, 2024.^[^ ^108^ ^]^	Human PBMC (simulated datasets from 1026 individuals)	Demuxalot	Genotype needed	0.906	3	93
		Vireo	Genotype free	0.885	7.2	24
		Dropulation	Genotype needed	0.884	7.5	12
		Souporcell	Genotype free	0.806	39.7	42
		Demuxlet	Genotype needed	0.751	3.8	98
		Freemuxlet	Genotype free	0.548	3.9	95
		ScSplit	Genotype free	0.292	236	58

The performance of genetic variation‐based demultiplexing hinges primarily on the genetic diversity between multiplexed samples, with a minimum SNP density (e.g., 0.2–0.34 SNPs k^−1^) required for algorithms to distinguish individuals reliably.^[^ ^110^ ^]^ Adequate sequencing depth is also essential to ensure robust detection of informative SNPs within sparse single‐cell data. The performance is also affected by doublet rates and the total number of pooled samples. The demultiplexing accuracy declines markedly as doublet rates rise, often more severely than when the number of pooled samples increases. Consequently, for experiments with high anticipated doublet rates, a hybrid approach that combines multiple demultiplexing methods is generally recommended to achieve the most accurate and robust results.^[^ ^108^, ^111^ ^]^

### 3.5 Hybrid Sample Demultiplexing

Innovative methodologies that integrate two or more of the above principles have been developed to enhance the accuracy, robustness, and applicability of sample demultiplexing (Figure 2E). For instance, Hedge is an ensemble tool that combines twelve different deconvolution methods, including both genetic and hashing‐based approaches. This integration improves cell recovery and efficiency, especially in noisy datasets.^[^ ^19^ ^]^ Similarly, HTOreader synergistically merges cell hashing with SNP analysis, using the genetic data to refine classification cutoffs and perform self‐validation, thereby improving cell recovery to ≈90%.^[^ ^18^ ^]^ Extending this integrative concept to scDNA‐seq, SNACS combines SNP and antibody marker data for demultiplexing, a strategy that achieves high sensitivity while providing visualization tools for quality control.^[^ ^112^ ^]^

## 4 Applications of Sample Multiplexing

### 4.1 Large‐Scale Clinical Cohort Studies

Sample multiplexing is a powerful strategy that enables cost‐effective, large‐scale single‐cell analyses from diverse biological sources while minimizing technical batch effects.^[^ ^83^, ^113^, ^114^, ^115^, ^116^ ^]^ Its applications have been particularly impactful in translational and clinical research.

**Figure 4:**
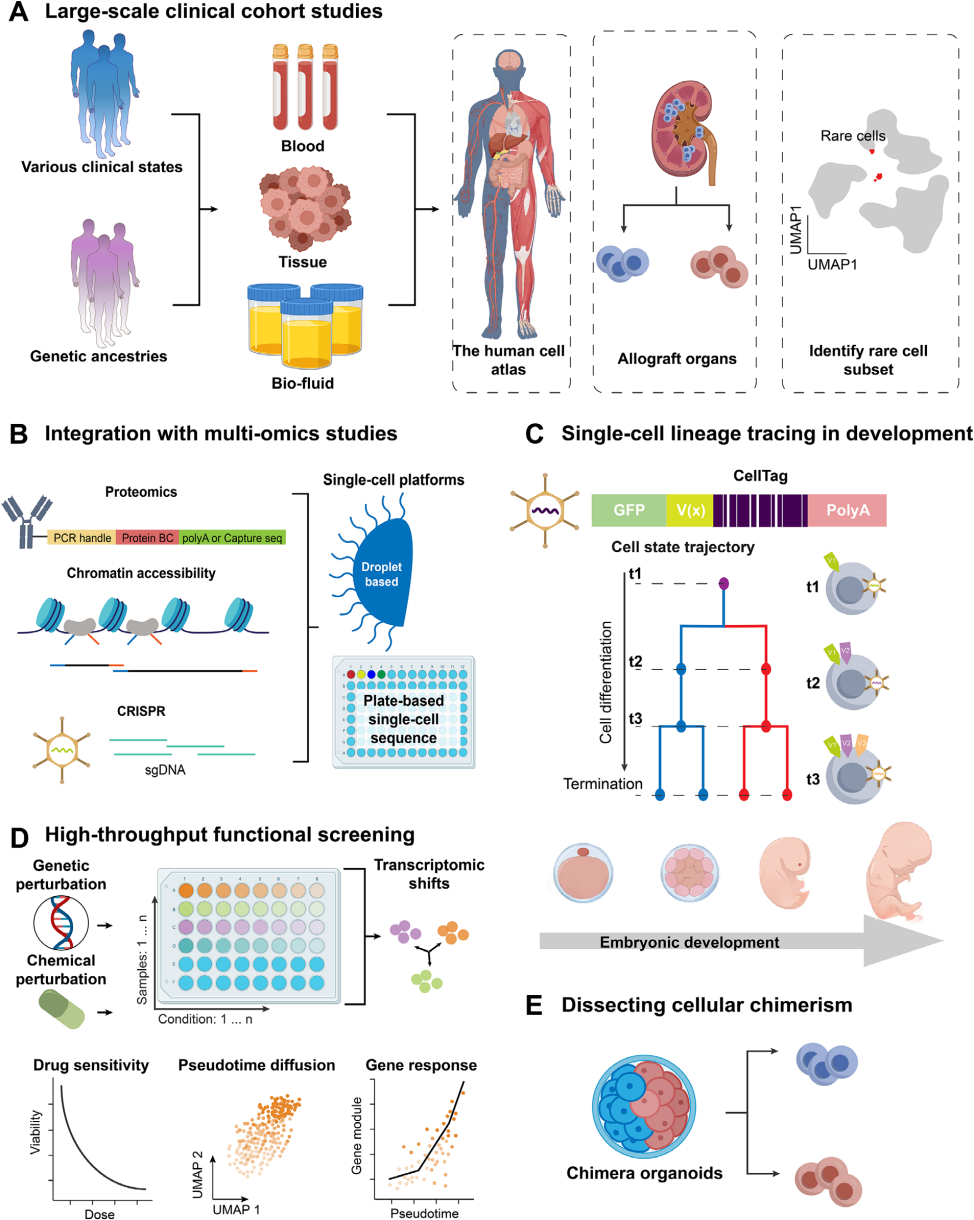
Applications of sample multiplexing for single‐cell sequencing. A) Sample multiplexing enables cost‐effective analysis across diverse disease states, treatment groups, or demographics. This has been instrumental in creating high‐resolution reference atlases and identifying rare cell subpopulations. B) The versatility of multiplexing is critical, enabling the simultaneous analysis of multiple molecular layers (e.g., transcriptome, proteome, epigenome) in clinical contexts. C) Timed cell tagging allows for the investigation of cell differentiation and lineage commitment in both in vivo and in vitro settings. D) Sample multiplexing facilitates high‐throughput screening of genetic or chemical perturbations at single‐cell resolution. E) Genetic variation‐based demultiplexing is a powerful tool for dissecting cellular chimerism, providing insights into the mechanisms of graft rejection and the development of organoids.

Multiplexing is essential for large‐scale patient cohort studies, as it allows for the simultaneous analysis of many samples, thereby reducing batch effects and enabling robust comparisons across disease states, treatment groups, or demographics (**Figure** **4**A). For example, genetic demultiplexing has been instrumental in creating high‐resolution single‐cell reference atlases from numerous healthy donors and patients.^[^ ^117^, ^118^, ^119^ ^]^ In clinical oncology, antibody‐based cell hashing was used to perform a comprehensive census of the bone marrow immune microenvironment in acute myeloid leukemia (AML),^[^ ^120^ ^]^ while multiplexed analyses of non‐small cell lung cancer (NSCLC) cohorts have refined tumor classification and improved patient stratification.^[^ ^121^ ^]^

A key benefit of multiplexing is its ability to increase sample size and cellular coverage, which is essential for identifying rare cell types critical to disease mechanisms. For example, the UDA‐seq method successfully identified scarce populations like age‐associated γδT cells and proteinuria‐associated podocytes from diverse clinical samples, even at frequencies below 0.5%.^[^ ^91^ ^]^ Similarly, leveraging natural genetic variation in transplant kidneys has enabled the detection of rare immune subsets, such as plasmacytoid dendritic cells and regulatory T cells, in the context of transplant rejection.^[^ ^122^ ^]^ This capability is vital for uncovering the subtle cellular dynamics that drive complex biological processes.

### 4.2 Integration with Multi‐Omics Studies

The versatility of multiplexing is critical for multi‐omics assays on samples like blood, biopsies, and cerebrospinal fluid to characterize complex biological systems (Figure 4B).

In oncology, multi‐omics single‐cell profiling provides unprecedented resolution of tumor ecosystems. For instance, in mixed phenotype acute leukemia (MPAL), it has been used to reveal inter‐ and intra‐tumoral transcriptional, immunophenotypic, and genetic landscapes in newly diagnosed patients.^[^ ^123^ ^]^ Similarly, multiplexed analysis of colorectal cancer patients has simultaneously profiled T‐cell transcriptomes, TCR sequences, and surface markers to identify prognostic and non‐prognostic T‐cell types.^[^ ^124^ ^]^

In immunology, multi‐omics helps dissect host immune responses to pathogens and evaluate disease‐driving cell populations. This has been demonstrated in detailed analyses of patient immune responses to COVID‐19.^[^ ^125^, ^126^ ^]^ In the context of autoimmune diseases, such as rheumatoid arthritis and lupus, multiplexing aids in identifying pathogenic cell subsets and evaluating drug efficacy at single‐cell resolution, thereby accelerating biomarker discovery and supporting personalized medicine strategies.^[^ ^127^, ^128^, ^129^ ^]^

In the field of organ transplantation, genetic demultiplexing enables multi‐omic profiling of mixed donor‐recipient cell populations. For example, it has been applied to bronchoalveolar lavage samples from lung transplant recipients to profile lung mesenchymal cells, providing critical insights into the cellular mechanisms of allograft dysfunction.^[^ ^130^ ^]^

### 4.3 Single‐Cell Lineage Tracing in Developmental Biology

Multiplexing techniques are invaluable for elucidating cell differentiation pathways and tracing lineages at single‐cell resolution, providing insights into developmental processes and disease progression.

High‐throughput multiplexed transcriptomics has been crucial for dissecting developmental hierarchies. In model organisms, it has been used to map mesodermal patterning in murine embryonic stem cells^[^ ^131^ ^]^ and to perform embryo‐scale reverse genetics in zebrafish, enabling the resolution of complex developmental trajectories (Figure 4C).^[^ ^132^ ^]^ This approach has been extended to human in vitro systems, such as cortical brain organoids, where multiplexing facilitates the longitudinal analysis of neurodevelopment and helps link genetic variations to specific phenotypes.^[^ ^133^ ^]^ Furthermore, in the context of glioblastoma, multiplexed single‐cell lineage tracing has been used to uncover the dynamics of drug resistance, allowing for the identification of molecular markers that predict resistant clones.^[^ ^134^ ^]^

### 4.4 High‐Throughput Functional Screening

Sample multiplexing enables the high‐throughput screening of genetic and chemical perturbations, allowing researchers to profile the transcriptional responses to thousands of perturbations simultaneously in a pooled format.^[^ ^135^, ^136^ ^]^ This capability is crucial for dissecting complex cellular responses, identifying cancer vulnerabilities, and elucidating therapeutic mechanisms (Figure 4D).

Multiplexing has transformed pharmaco‐transcriptomic profiling by enabling the analysis of drug responses at single‐cell resolution across many conditions. For instance, live‐cell barcoding with antibody‐oligonucleotide conjugates has been used to test patient‐derived tumor samples against various drugs,^[^ ^137^ ^]^ while other studies have conducted large‐scale kinase inhibitor screens.^[^ ^138^ ^]^ Platforms like MIX‐Seq facilitate these applications by supporting multiplexed profiling of both drug and CRISPR perturbations in cancer cell lines.^[^ ^139^ ^]^

For massively parallel chemical screening, sci‐Plex allows for the quantification of global transcriptional responses to thousands of compounds at single‐cell resolution, providing deep insights into drug mechanisms and intercellular heterogeneity.^[^ ^83^ ^]^ This has been extended by sci‐Plex‐GxE, which supports combined single‐cell genetic and chemical screening to define kinase dependencies in cancer drug responses.^[^ ^140^ ^]^

By combining multiplexed single‐cell RNA‐seq with pooled CRISPR screens, researchers can systematically link genetic perturbations to their transcriptional consequences. Perturb‐seq is a foundational platform in this area, used to dissect complex pathways such as the unfolded protein response by revealing functional gene clustering and signaling interactions.^[^ ^141^ ^]^ The versatility of this approach is demonstrated by adaptations like CPA‐Perturb‐seq, which has enabled the multiplexed characterization of alternative polyadenylation regulators.^[^ ^142^ ^]^

### 4.5 Dissecting Cellular Chimerism

In solid organ transplantation, genetic variation‐based demultiplexing provides critical insights into cellular chimerism—the mixture of donor and recipient cells—and its role in graft rejection. By leveraging genetic differences, such as recipient‐donor sex mismatches or single‐nucleotide variants (SNVs), researchers can precisely determine the origin of each cell within a transplanted organ. This has been used to define leukocyte chimerism in kidney transplants^[^ ^143^ ^]^ and to reveal that a significant fraction of fibroblasts in failing allografts were recipient‐derived, implicating them in the fibrotic process.^[^ ^144^ ^]^ These applications underscore the unique strength of genetic demultiplexing for dissecting cellular contributions in mixed, genetically distinct populations, even in challenging clinical samples.

Another notable application is the creation of “Chimeroids”—genetically chimeric organoids formed by mixing cells from different human donors.^[^ ^145^ ^]^ By using demultiplexing tools like Demuxlet, researchers can efficiently deconvolve the cellular identities within these chimeric brain organoids. This innovative approach provides a scalable system to elucidate how genetic variance influences normal brain development and contributes to disease phenotypes^[^ ^146^ ^]^ (Figure 4E).

## 5 Methodologies Comparison and Selection

### 5.1 Comparison of Sample Multiplexing Strategies

Antibody‐based sample labeling is widely adopted due to its simplicity, commercial availability, and proven efficacy for robust cell types like human PBMCs and established cell lines. However, its primary limitation is the dependence on sufficient surface antigen expression, which can lead to poor performance on certain fragile cell types (e.g., mouse embryonic brain cells) or on isolated nuclei where surface markers are absent.^[^ ^70^, ^147^ ^]^

For challenging samples, including fragile cells or isolated nuclei, lipid‐based methods (e.g., MULTI‐seq, CellPlex) provide a superior alternative.^[^ ^10^, ^69^ ^]^ By directly anchoring barcodes into the lipid membrane, this approach bypasses the need for specific antigens. It excels in nuclear labeling and is robust for demultiplexing snATAC‐seq data and handling frozen samples where whole‐cell integrity may be compromised.^[^ ^70^ ^]^

**Figure 5:**
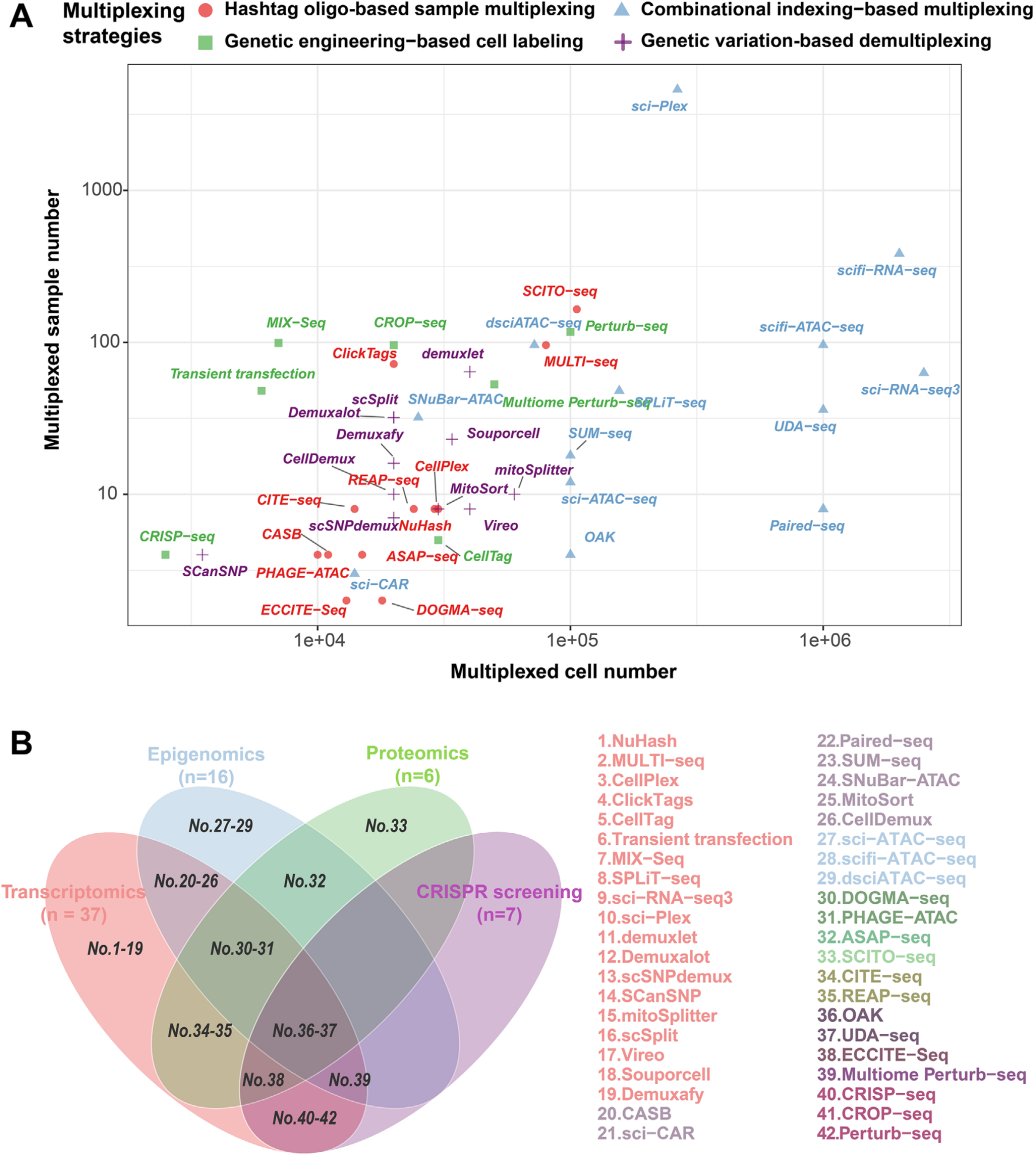
Scalability and multi‐omics compatibility of sample multiplexing strategies. A) A scatter plot illustrating the multiplexing capacity of various single‐cell strategies, comparing the number of multiplexed cells (x‐axis) against the number of multiplexed samples (y‐axis). Each point represents a specific method, with its color denoting the corresponding methodological category. B) A Venn diagram illustrating the multi‐omics capabilities of different methods. Each circle represents a major modality (Transcriptomics, Epigenomics, Proteomics, and CRISPR screening), with overlapping regions identifying techniques that support simultaneous multi‐modal analysis.

Combinatorial indexing methods are revolutionizing single‐cell genomics due to their exceptional scalability, supporting the multiplexing of hundreds to thousands of samples^[^ ^83^ ^]^ (**Figure** **5**). By sequentially barcoding fixed nuclei through “split‐pool” steps, these techniques enable comprehensive multi‐omic profiling without the need for specific labeling reagents. Although the experimental protocols can be time‐consuming and are primarily restricted to fixed cells or nuclei, combinatorial indexing offers a powerful and cost‐effective solution for large‐scale single‐cell research.^[^ ^147^ ^]^

Genetic engineering offers a cost‐effective and highly accurate method for cell labeling, particularly for analyzing cellular responses to drug perturbations in cell lines. However, its reliance on viral vectors, complex workflows, and often low transduction efficiency in primary cells severely restricts its applicability in clinical contexts or with fragile samples.

Leveraging endogenous genetic variation (e.g., SNPs) provides a label‐free strategy for demultiplexing that minimizes cellular perturbation by eliminating external labeling and washing steps. This is highly advantageous for fragile cells or nuclei, which can suffer substantial loss (≈20–30%) during processing.^[^ ^148^ ^]^ The method achieves high accuracy (>90%) across diverse tissues and species.^[^ ^149^ ^]^ Its main limitations are the requirement for sufficient genetic diversity between samples (a minimum of 0.2–0.34 SNPs kb^−1^), making it unsuitable for inbred organisms, and its demand for significant computational resources.^[^ ^110^ ^]^

Hybrid methods, which integrate complementary strategies such as cell hashing and SNP‐based demultiplexing, can further enhance performance. While these approaches may increase experimental and computational complexity, they often surpass single‐method techniques in both accuracy and robustness.^[^ ^18^, ^19^ ^]^

### 5.2 A Practical Guide for Method Selection

**Figure 6:**
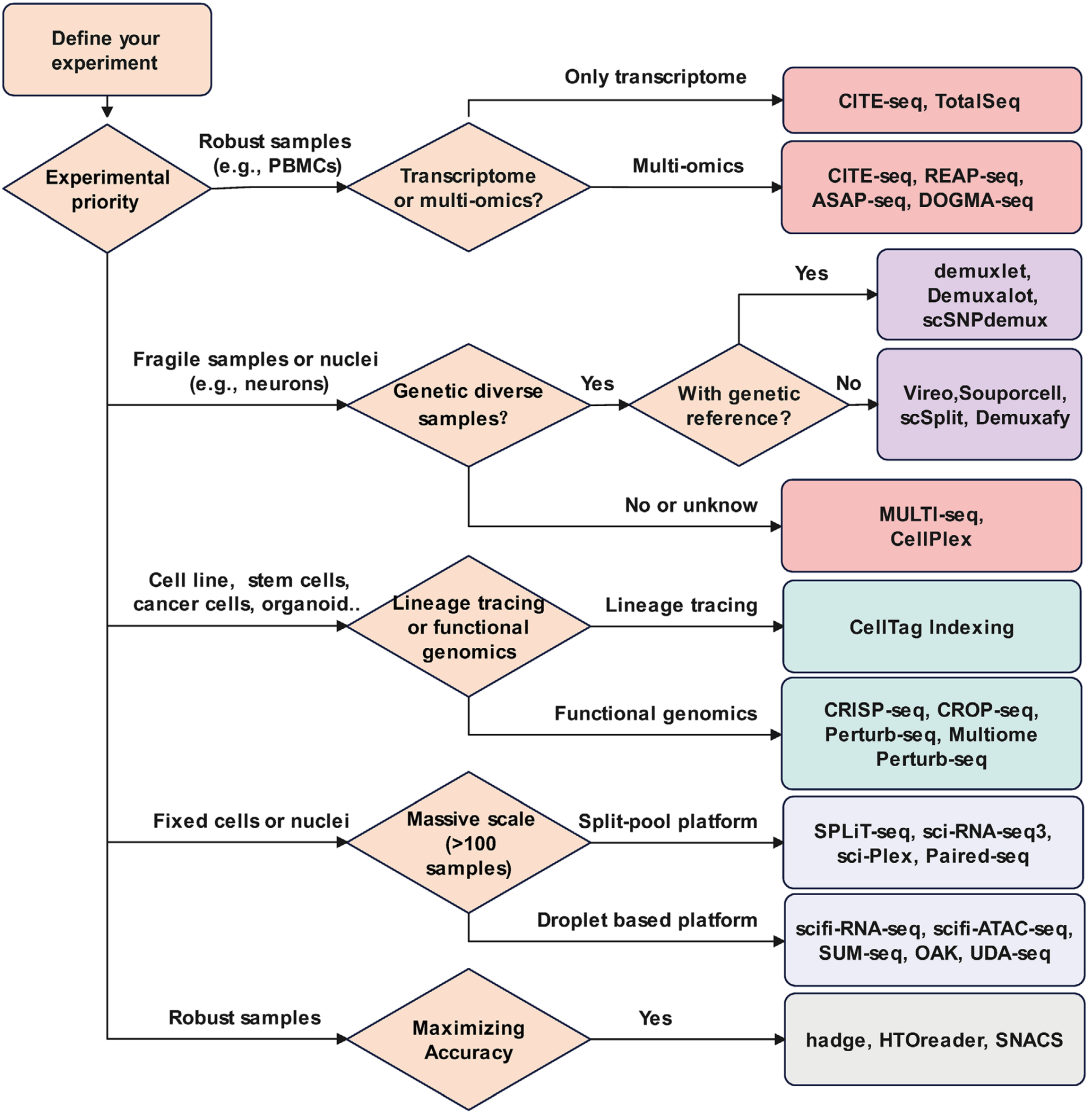
A decision‐making flowchart for selecting a sample multiplexing strategy. This flowchart provides a practical guide for selecting the most appropriate single‐cell multiplexing method based on key experimental considerations. The decision path is determined by several key factors, including the nature of the sample (e.g., robust cells versus fragile nuclei), the primary scientific objective (e.g., lineage tracing, functional genomics, or the need to maximize accuracy). The diagram highlights representative methods for each scenario and is not intended to be an exhaustive list; for a more comprehensive overview, please refer to Table S1 (Supporting Information).

Selecting an appropriate sample multiplexing method requires balancing several key factors, including multiplexing capacity, sample type compatibility, cost per sample, and multi‐omics capabilities (**Figure** **6**; **Table** **5**).

**Table 5:** Comparisons of multiplexing strategies for single‐cell sequencing.

Multiplexing Strategy	Multiplexing capacity	Estimated cost per sample	Application scenario	Advantages	Challenges
Antibody‐based sample labeling	Low (≈8–16 plex)	≈$350	Standard applications with robust cells (e.g., PBMCs).	Simple, commercially available, and streamlined workflow.	Requires sufficient surface protein expression; epitope integrity is crucial.
Lipid‐based sample labeling	Medium (≈12–96 plex)	≈$200	Fragile cells and isolated nuclei (e.g., brain, frozen tissue).	Excellent for fragile cells and nuclei; independent of surface proteins.	Lower specificity can generate high background noise.
Combinatorial indexing ‐based multiplexing	Very High (100 to 1000 plex)	<$25	Massive‐scale experiments or multi‐omics studies.	Unparalleled scalability and cost‐effective flexibility.	Time‐consuming wet‐lab protocols require fixed cells or nuclei.
Genetic engineering‐based cell labeling	Variable (≈5–96 plex)	Highly variable	Lineage tracing or functional screens in cell lines.	Provides a permanent, heritable barcode for tracing the lineage of live cells.	Complex (viral vectors, biosafety); low transduction efficiency in primary cells.
Genetic variation‐based demultiplexing	Medium (≈2–64 plex)	≈$250	Clinical cohorts or precious samples where labeling is undesirable.	Label‐free method that maximizes cell retention by eliminating the need for washing steps.	Requires significant genetic diversity between samples; computationally intensive.
Hybrid Methods	Variable	Unknown	Experiments demanding the highest possible accuracy.	Achieves the highest accuracy and robustness by combining methods.	Increased experimental and computational complexity.

For standard applications involving robust cells such as PBMCs and cell lines, antibody‐based sample labeling is often the preferred choice due to its simplicity, commercial availability, and streamlined workflow. However, for fragile cells or isolated nuclei—such as those from brain tissue or frozen samples—lipid‐based methods offer a superior alternative, as they are gentler on membranes and do not depend on protein epitopes. For multiplexing samples with diverse genetic backgrounds, particularly in clinical cohorts, leveraging endogenous genetic variation is the recommended strategy. This label‐free approach eliminates upstream labeling and washing steps, thereby maximizing cell retention and often improving cell recovery rates compared to external tagging methods.^[^ ^147^ ^]^

Large‐scale functional genomics or lineage‐tracing studies may utilize genetic engineering–based labeling, which provides a permanent, heritable barcode. This approach is ideally suited for linking CRISPR‐based perturbations to transcriptional readouts, although it requires more elaborate experimental protocols. For applications demanding massive scale or multi‐omics integration, combinatorial indexing offers unparalleled scalability and flexibility cost‐effectively, albeit at the cost of more time‐intensive wet‐lab procedures.

Ultimately, for studies requiring the highest level of accuracy, hybrid strategies that combine complementary approaches—such as cell hashing and genetic variants—often deliver superior demultiplexing robustness, albeit at the expense of increased experimental and computational complexity.

## 6 Conclusion

Sample multiplexing is a powerful technique that has become central to high‐throughput genomics, particularly for next‐generation and single‐cell sequencing. By enabling the pooling of multiple samples into a single experimental run, multiplexing substantially increases throughput, lowers per‐sample costs, and minimizes technical batch effects. Furthermore, it improves data quality by allowing for the robust computational identification and removal of multiplets. These advantages have facilitated large‐scale cohort studies, the elucidation of cellular differentiation pathways, high‐throughput functional screens, and integrated multi‐omics analyses of complex biological systems.^[^ ^150^ ^]^

Despite its advancements, single‐cell multiplexing confronts several persistent challenges. A fundamental trade‐off exists between the number of multiplexed samples and the sequencing depth per cell. High‐plex experiments can compromise the detection of rare biological signals if sequencing resources are spread too thin. The high initial cost of specialized barcoding reagents and the need for substantial bioinformatics expertise can be significant barriers for many laboratories. Moreover, managing doublet rates is a critical concern, as higher levels of multiplexing require sophisticated computational methods for accurate removal.^[^ ^151^, ^152^, ^153^ ^]^ Ensuring robust performance across diverse and challenging sample types, such as heterogeneous tissues or low‐input clinical specimens, remains a significant hurdle that can impact demultiplexing accuracy.^[^ ^154^, ^155^ ^]^ The lack of standardized benchmarking protocols makes direct and unbiased comparisons between different multiplexing methods difficult.

As a rapidly evolving field, single‐cell multiplexing presents several key areas that require future innovation to overcome existing limitations. A vital priority is to accelerate clinical translation by developing robust protocols for challenging samples, such as formalin‐fixed paraffin‐embedded (FFPE) tissues and low‐cell‐input biopsies.^[^ ^64^ ^]^ Technologically, a key frontier is the integration of deeper multi‐omic and spatial data, combining readouts from modalities like metabolomics with spatial information to elucidate cellular function in the native tissue context.^[^ ^150^ ^]^ These experimental advances necessitate the parallel development of smarter, user‐friendly computational algorithms capable of handling the resulting complexity, ensuring robust demultiplexing and clear interpretability even from noisy datasets.^[^ ^156^, ^157^ ^]^ Collectively, these advancements are essential for accelerating biomarker discovery, improving clinical diagnostics, and enabling AI‐driven personalized therapies.^[^ ^158^, ^159^ ^]^

## Conflict of Interest

The authors declare no conflict of interest.

## Author Contributions

W.C., Y.G., and W.H. performed conceptualization. Y.G. performed visualization. Y.G. wrote the original draft. W.H., W.Y., and W.C. wrote, reviewed and edited the final manuscript.

## Supporting information



Supporting Information

## Data Availability

No new data were generated in support of this review.
